# Optimization of a siRNA Carrier Modified with a pH-Sensitive Cationic Lipid and a Cyclic RGD Peptide for Efficiently Targeting Tumor Endothelial Cells

**DOI:** 10.3390/pharmaceutics7030320

**Published:** 2015-09-14

**Authors:** Tomoya Hada, Yu Sakurai, Hideyoshi Harashima

**Affiliations:** Laboratory for Innovative Nanomedicine, Faculty of Pharmaceutical Sciences, Hokkaido University, Kita-12, Nishi-6, Kita-ku, Sapporo 060-0812, Japan; E-Mails: tmy-1011@mail.sci.hokudai.ac.jp (T.H.), yu-m@pharm.hokudai.ac.jp (Y.S.)

**Keywords:** siRNA delivery, tumor endothelial cells (TECs), pH-sensitive carrier, cyclic RGD, post-modification method

## Abstract

In recent years, anti-angiogenic therapy has attracted much interest because it is a versatile approach to treating most types of tumors, and therefore would be expected to be applicable for various cancers. Severe adverse events in patients treated with currently available anti-angiogenic therapeutics have, however, been reported, and these are caused by their inhibitory effects in normal tissue. To achieve an efficient anti-angiogenic therapy with minimal toxicity, a drug delivery system (DDS) specific to tumor endothelial cells (TECs) is needed. Cyclic RGD (cRGD) is a well-known ligand against α_V_β_3_ integrin that is expressed at high levels in the cell surface of TECs. To address this issue, we previously developed a cyclic RGD-equipped liposomal DDS (RGD-MEND) in which small interfering RNA (siRNA) was encapsulated. However, in the previous study, details of the preparation steps were not thoroughly examined. In this paper, to produce the most efficient delivery of therapeutic TECs, we explored optimum preparation conditions and components of the RGD-MEND. The cellular uptake and silencing ability of the RGD-MEND were investigated as a function of ligand density, poly(ethyleneglycol) linker length, and lipid composition. As a result, a knockdown efficiency that was five-fold higher than that of the previously reported one (ED_50_, from 4.0 to 0.75 mg/kg) was achieved.

## 1. Introduction

Since angiogenesis plays a pivotal role in cancer progression and metastasis, anti-angiogenic therapeutics are thought to be attractive for the development of new types of cancer therapy [1]. New vessel formation occurs when tumor tissue grows over 2 mm^3^ [2] and, therefore, anti-angiogenic therapy would be expected to be a new class of medicines for many types of cancers because of the universality of angiogenesis. In addition, anti-angiogenic therapeutics are relatively safe compared to traditional anti-cancer drugs, since they do not focus on cell-killing effects. Several anti-angiogenic therapeutics, including multi-kinase inhibitors and an antibody against vascular endothelial cell growth factor (VEGF), have now been approved [3]. However, since VEGF cascades are involved in maintaining homeostasis for the entire body, recent studies revealed that the somatic inhibition of the VEGF cascade could induce severe adverse effects such as hypertension, bowel perforation, and arterial thromboembolisms [4,5]. For these reasons, tumor endothelial cell (TEC)-specific anti-angiogenic therapeutics are needed to achieve safe, effective cancer therapy.

To develop a new anti-angiogenic therapy without adverse effects, we attempted to combine a TEC-specific drug delivery system (DDS) and small interfering RNA (siRNA), which inhibits mRNA expression in a sequnce-specific manner. Recent studies revealed that mRNA expression in TECs is quite different from that of normal endothelial cells [6]. Therefore, if genes related to abnormal angiogenesis in tumor tissue could be specifically suppressed, it might be possible to circumvent adverse effects caused by the unnecessary inhibition of newly identified genes in normal tissue. However, developing such a specific inhibitor against such genes would be time-consuming and costly. In contrast, siRNA is very compatible due to sequence-specific inhibition, once a siRNA DDS is developed [6]. Due to a huge, hydrophilic molecule and susceptibility to ribonuclease, a drug delivery system needs to be developed for realizing siRNA medicine. We previously reported that a lipid nanoparticle (multi-envelope-type nanodevice: MEND) containing a pH-sensitive lipid, YSK05 (YSK-MEND), could efficiently be delivered to target cells [7]. In the previous study, 2-(*p*-Toluidino)-6-naphthalenesulfonic acid (TNS) assay, which is a fluorescent molecule only in a lipophilic and cationic environment, revealed that the acid dissociation constant (pK_a_) of YSK05 was approximately 6.5 [7]. Furthermore, YSK05 containing lipid nanoparticles exhibited YSK05-induced membrane fusion in a pH-dependent manner, even in the presence of ligand modification [8]. We also developed a TEC-specific delivery system, the RGD-MEND, which was modified with a TEC-targeting ligand, cyclic RGD (cRGD). A target receptor of cRGD is α_V_β_3_ integrin, which is specifically and highly expressed in TECs and some types of cancer cells [9]. Inhibition of the VEGF receptor 2 (VEGFR2), which is a dominant receptor in the angiogenesis of tumor tissue, by the systemic injection of the RGD-MEND into tumor-bearing mice resulted in tumor regression via an anti-angiogenic effect. Despite the success of *in vivo* knockdown, very large amounts of siRNA (4.0 mg/kg) were needed to inhibit the TECs’ mRNA.

The aim of this study was to decrease the dose needed to inhibit the mRNA of TECs by half (ED_50_). To accomplish this, we optimized the parameters for preparing the RGD-MEND to improve the efficiency of the *in vivo* knockdown in terms of ligand modification. We previously developed two types of MENDs that contained different lipid compositions for use in *in vitro* knockdown (MEND_vitro_) [7] and *in vivo* hepatocyte knockdown (MEND_vivo_) [10,11]. The procedure used to prepare the RGD-MEND involved three steps; base MEND assembly, RGD post-modification, and purification. Although the MEND_vivo_ was expected to show a higher gene knockdown in TECs than the MEND_vitro_, it was not possible to modify the MEND_vivo_ with the RGD ligand under the same conditions that were used for the MEND_vitro_ (60 °C, 30 min, in water). Although we are not sure why ligand modification resulted in failure, the absence of a helper lipid in the composition of the MEND_vivo_ might affect its insertion efficiency. Other groups reported that the addition of ethanol destabilized the lipid envelope [12] and the PEG-lipid was modified to a lipid nanoparticle in ethanol solution [2]. Based on these studies, we examined this method for facilitating the efficiency of modifying the ligand to the MEND_vivo_.

## 2. Experimental Section

### 2.1. Materials

YSK05 was synthesized as previously described [7]. Cholesterol (chol) and RPMI-1640 medium were obtained from Sigma–Aldrich (St. Louis, MO, USA). Primers and siRNAs were purchased from Hokkaido System Science Co., Ltd. (Sapporo, Japan), and their sequences are listed in Table S1. 1,2-dimyrstoyl-*sn*-grycero, poly(ethylene glycol)_2000_ (PEG-DMG), 1,2-distearoyl-*sn*-grycero, poly(ethylene glycol)_2000_ (PEG-DSG), 1,2-distearoyl-*sn*-grycerophosphoethanolamine, *N*-hydroxy-succinimide poly(ethylene glycol)_2000, 3400 and 5000_ (NHS-PEG_2k_-DSPE, NHS-PEG_3.4k_-DSPE, NHS-PEG_5k_-DSPE), and 1-palmitoyl-2-oleoyl-*sn*-grycerophosphoethanol amine (POPE) were obtained from NOF CORPORATION (Tokyo, Japan). *Griffonia simplicifolia* isolectin B4 (GSIB4) was purchased from VECTOR Laboratories (Burlingame, CA, USA). Fluorescently hydrophobic molecules DiI and DiD were purchased from Life Technologies Corp. (Carlsbad, CA, USA). Cyclic RGD (cyclo(Arg-Gly-Asn-d-Phe-Lys), cRGD) was obtained from Peptides International, Inc. (Louisville, KY, USA). Dialysis membranes Spectra/Por 6 (MWCO 1000) were obtained from Spectrum Labs (Rancho Dominguez, CA, USA). All other chemicals used in the study were commercially available ones.

### 2.2. Synthesis of Ligand Conjugated PEG-Lipid

To display a cyclic RGD on the surface of the liposomal nanocarrier, cRGD was conjugated to the head of the PEG-lipid via an amide bond, as previously reported [8]. Briefly, NHS-PEG-DSPE and the free cyclic RGD peptide (1.1 equiv.) were incubated in PBS containing potassium and magnesium ions (PBS (−)) for 24 h at room temperature. The resulting mixture was subjected to dialysis against PBS (−) for 1 h, and against deionized distilled water (DDW) for 2 and 24 h. The dialysate was freeze-dried to give a white powder (RGD-PEGs). The white powder was dissolved in ethanol (EtOH) and preserved at −80 °C until used in experiments. Three types of PEG linkers (M.W. 2000, 3400 and 5000) were used in the synthesis, and referred to as PEG_2k_-RGD, PEG_3.4k_-RGD, PEG_5k_-RGD. The synthesis of these PEG-lipids were confirmed by matrix-assisted laser desorption ionization time-of-flight mass spectrometry (Figure S1).

### 2.3. Preparation of MENDs

The MEND was prepared by the tertiary butanol (*t*-BuOH) dilution method as previously reported [10,13]. The lipids were dissolved in 400 μL of *t*-BuOH at 7.5 mM, and siRNA was dissolved in 200 μL of 2 mM citric buffer (pH 4.0) in another tube. The siRNA solution was then gradually added to the lipid solution under vigorous mixing. The resulting mixture was then immediately diluted with 4.0 mL of PBS (−), and was then subjected to ultrafiltration with Vivaspin (MWCO 100,000 Da) twice to remove free siRNA molecules and *t*-BuOH from the preparation. If the MEND was fluorescently labeled, DiO, DiI, or DiD was added to the lipid solution before mixing with the siRNA solution at 0.5 mol% of total lipid. When the MENDs were modified with PEG-lipids, the RGD-MEND and specified amounts of PEG-lipids were incubated in 5 mM citric buffer (pH 5.0). Ethanol was added to the mixture under mixing to the indicated concentrations for 15–60 min at 30–60 °C. The MENDs were characterized by a dynamic light scattering method with a Zetasizer Nano ZS ZEN3600 instrument (Malvern Instruments, Worcestershire, UK). The encapsulation efficiency (EE) and recovery rate (RR) of siRNA were determined using RNA quantification kit, RiboGreen (Life Technologies), as previously performed [8]. When the diameter needed to be increased, PEG-DMG was decreased from 3 mol% (small) to 1 mol% (medium) and 0.3 mol% (large).

### 2.4. Cell Culture

OS-RC-2 cells (human renal cell carcinomas) and 4T1 cells (murine breast cancer) were obtained from the American Type Culture Collection. These cells were maintained in RPMI-1640 medium supplemented with 10% fetal bovine serum, 100 U/mL of penicillin, and 100 μg/mL of streptomycin at 37 °C in a 5% CO_2_ humidified atmosphere. Cells were passed with 0.05% trypsin, 0.55 mM ethylenediaminetetraacetic acid in PBS (−) when 70%–80% confluent.

### 2.5. Measurement of Cellular Uptake

To measure the ligand ability of the RGD-MENDs, 1.0 × 10^5^ OS-RC-2 cells were plated onto six-well plates (Corning, Corning, NY, USA) 24 h before the cellular uptake experiment. Fluorescent labeled MENDs were added to the wells at 24.4 μM (lipid), in which the lipid concentration was converted to approximately 100 nM of the siRNA concentration. The amount DiO or DiD-labeled MENDs added was adjusted to the sample showing the maximum FI by measuring the FI of each samples with Spectra MAX (Molecular Devices Corp., Menlo Park, CA, USA). After a 2-h incubation, cells were trypsinized, and the preparation then centrifuged (700× *g*, 3 min, 4 °C). Supernatant was removed, and 500 μL of FACS buffer (0.5% bovine serum albumin, 0.01% sodium azide in PBS (−)) was added to the cell pellet. After the centrifugation (700× *g*, 3 min, 4 °C), 500 μL of FACS buffer was added. This procedure was repeated twice to remove cell debris and unbound MENDs. Finally, the cell pellet was suspended in 1 mL of FACS buffer, and was used as a measurement sample. Samples were measured with FACSCalibur (Becton Dickinson, Franklin Lakes, NJ, USA). Data were analyzed by Cell Quest (Becton Dickinson).

### 2.6. In Vitro Knockdown Experiment

To evaluate the knockdown efficacy of the MENDs, 1.0 × 10^5^ OS-RC-2 cells were plated on six-well plates (Corning Inc., Corning, NY, USA) 24 h before the experiment. Cells were transfected with indicated siRNA concentration of MEND for 24 h. Then, cells were lysed with 350 μL of TRIzol (Life Technologies Corp.), and then total RNA was extracted from cell lysates according to the manufacture’s protocol. For converting mRNA to cDNA, 1.0 μg of mRNA was reverse-transcribed with High Capacity RNA-to-cDNA kit (Life Technologies Corp.) as previously reported [14]. The cDNA was preserved in −20 °C until the PCR experiment.

### 2.7. Animal Experiments

Balb/c mice (female, four-week-old) and ICR mice (male, four-week-old) were obtained from Japan SLC (Shizuoka, Japan). Balb/c nude mice (male, four-week-old) were purchased from Japan CLEA (Tokyo, Japan). To prepare tumor-bearing mice, Balb/c (for 4T1) and Balb/c nude mice (for OS-RC-2) were anesthetized with diethylether, and the right flanks of the mice were inoculated with 1.0 × 10^6^ cells. When tumor volume reached 100–200 mm^3^, the mice were used for *in vivo* experiments. These procedures were reviewed and approved by the Hokkaido University Animal Care Committee in accordance with the Guide for the Care and Use of Laboratory Animals (approved number: 13-0183 on 10 March 2015). For knockdown experiments, the MENDs were systemically injected into mice via the tail vein at the indicated dosages, and, after 24 h, tissue was excised and minced. TRIzol reagent (500 μL) was added to the minced tissue, and the tube of the TRIzol reagent was vigorously shaken by PreCellys (Bertin Technologies, Montigny-Le-Bretonneux, France). The following procedure for preparing cDNA was the same as that for the *in vitro* cultured cells.

### 2.8. Quantification of mRNA Expression Level with Quantitative Reverse Transcription Polymerase Chain Reaction (qRT-PCR)

To determine the mRNA expression with qRT-PCR, 5.0 μL of 50-fold diluted cDNA was mixed with 0.075 μL of forward and reverse primes and 7.5 μL of THUNDIRBIRD qPCR Mix (TOYOBO LIFE SCIENCE, Osaka, Japan) and 2.35 μL of DDW (total 15.0 μL). The mixture was then subjected to PCR with Light Cycler-480 (Roche Diagnostics, Germany) according to the manufactures’ protocol. The expression level was calculated with ΔΔ*C*_t_ method. All primers used in this manuscript were investigated in an amplification efficiency, and the sequences are listed in Table S1.

### 2.9. Measurement of Tumor Accumulation of MENDs

Tumor accumulation was evaluated with fluorescent labeled MENDs. The DiI-labeled MEND was administered to tumor-bearing mice via the tail vein. Tumor tissue was collected 24 h after the administration, and the tumor tissues were homogenized in 50 mg tissue/mL 1% SDS by PreCellys. The homogenates were then centrifuged (15000× *g*, 5 min, 4 °C), and the fluorescence intensity of the homogenates was measured with a plate reader, SpectraMax Paradigm (Molecular Devices, Tokyo, Japan, excitation/emission 520/565 nm). Standard curve was prepared by adding a known quantity of the fluorescent labeled MEND to the homogenate from the non-treatment.

### 2.10. In Vivo Observation of MENDs with Con-Focal Laser Scanning Microscopy (CLSM)

To assess the intratumoral distribution of RGD-MENDs, CLSM observation was conducted for tumor-bearing mice (OS-RC-2). These mice were injected with the DiD-labeled MENDs at a dose of 1.0 mg/kg. Forty microgram of FITC-labeled GSIB4 was injected via the tail vein 10 min before collecting the tumor tissue. The excised tumor tissue was cut into the half, and the specimens were observed using an A1 confocal imaging system (Nikon, Tokyo, Japan).

## 3. Results and Discussion

### 3.1. Optimization of Ligand Modification Method

**Figure 1:**
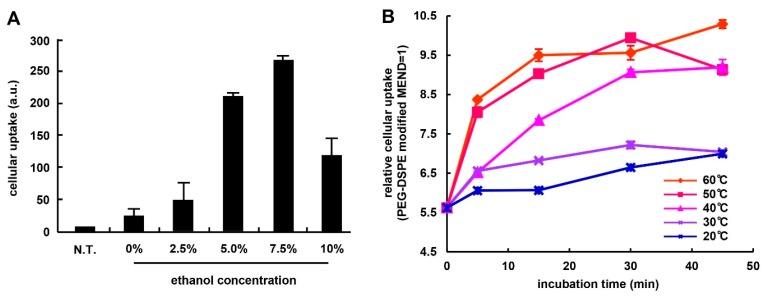
(**A**) The effect of ethanol concentration on modifying the ligand on the surface of the MENDs. MENDs were incubated with the ligand derivatives in citric buffer (pH 5.0) containing ethanol 0–10% at 45 °C for 20 min. The amount of cellular uptake of the MENDs was evaluated by flow cytometry (FCM). Data represents mean ± S.D. (**B**) The effect of incubation temperature in the post-modification step. When the temperature and incubation time were varied from 20 (blue), 30 (purple), 40 (pink), 50 (orange), and 60 °C (red), and 5–45 min, respectively, the cellular uptake of RGD-MENDs at 2 h after the transfection was evaluated by flow cytometry. Data represents mean ± S.E.M.

Optimal conditions for modifying the MENDs with a ligand were explored with a focus on ethanol concentration and the incubation conditions for the MEND_vivo_. The lipid compositions of these two carriers are shown in Table 1. In the study, OS-RC-2 cells (human renal cell carcinoma cells) were used as a α_V_β_3_ integrin-positive cell line (Figure S2). We assessed the effect of ethanol concentration (0–10% *v*/*v*: ethanol (EtOH)/water) on facilitating the cellular uptake of MENDs by ligand modification. The cellular uptake amount was increased up to a 7.5 mol% modification (Figure 1A). In contrast, a 10 mol% modification decreased the cellular uptake of the MEND. In a previous report, the effect of ethanol on the membrane stability of preformed liposomes was found to be decreased and, hence, protein was incorporated into liposomes by incubation in the presence of ethanol [15]. Herein, ligand-PEG was engrafted onto the MEND based on a similar mechanism. Moreover, a more recent report revealed that the efficiency of post-modification was dependent on the incubation time and temperature [16]. Therefore, this result is consistent with the previous other reports.

**Figure 2:**
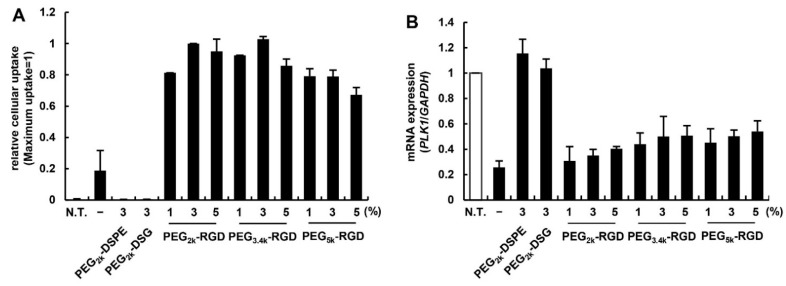
The effect of linker derivatives on the *in vivo* siRNA delivery. (**A**) The effect of the length of the PEG-linker and the density of RGD PEG-lipids on the uptake of MENDs. The cellular uptakes of RGD-MENDs were measured by FCM 2 h after the transfection of fluorescently labeled MENDs. Data represents mean ± S.D. (*n* = 3). (**B**) The effect of the linker length and density on the gene silencing efficiency of MENDs. Gene silencing was evaluated as a decrease of polo-like kinase 1 (*PLK1*) mRNA level estimated with qRT-PCR. Data represents mean ± S.D. (*n* = 3).

Accordingly, pre-formed MEND_vivo_ (denoted as “–” in Figure 2A,B) showed a robust silencing. In the RGD-modified MENDs, the gene silencing efficiency seemed to decrease as the linker length and density became larger. This decrease can be attributed to the additive effect of the increased uptake by RGD (Figure 2A) and the inhibition of endosomal escape by PEGylation accompanied by ligand density. Judging from these results, we assumed that 3 mol% of PEG_2k_-RGD modification was an optimal amount for the ligand modification for MEND_vivo_.

**Table 1:** Lipid composition of MEND_vivo_ and MEND_vitro_.

Carrier Name	Lipid Composition	Ref.
MEND_vivo_	YSK05/POPE/chol (50/25/25) + 3 mol% PEG-DMG	[7]
MEND_vitro_	YSK05/chol (70/30) + 3 mol% PEG-DMG	[11,14]

POPE: 1-palmitoyl-2-oleoyl-*sn*-glycerophosphoethanol amine; chol: cholesterol.

### 3.2. Effect of PEG Linker and Ligand Density on in Vitro siRNA Delivery Using MEND_vivo_

We then evaluated the impact of the length of the PEG linker and the ligand density on cellular uptake and the gene knockdown efficacy of the RGD-MENDs. The amount of RGD-MENDs modified with 2000–5000 length and 1–5 mol% of RGD PEG-lipids that were internalized was measured by flow cytometry (FCM) (Figure 2A). Non-ligand conjugated PEGs (PEG_2k_-DSPE and PEG_2k_-DSG) were regarded as a negative control. The extent of RGD-modification increased the cellular uptake of MENDs by three- to five-fold. Of these, the PEG_5k_-linker was generally inferior to the others in ligand ability. We next assessed the relationship between the gene silencing efficacy and ligand-PEG structure. Similarly, when the PEG-linker and ligand ratio was varied, the mRNA level of polo-like kinase 1 (*PLK1*) was determined by qRT-PCR (Figure 2B). In the case of negative controls (PEG_2k_-DSPE and PEG_2k_-DSG), no gene silencing was observed. This weak silencing can be attributed to the inhibition of endosomal escape by PEGylation, in addition to a low cellular uptake of these carriers. Although the pre-formed MEND included PEG-DMG (C14-anchor), the PEG-lipid with the C14-anchor was rapidly removed from the surface of liposomes [17] and, hence, a PEG-lipid containing a short anchor could not inhibit cellular uptake and the endosomal escape of nanocarriers greatly.

### 3.3. Effect of PEG-Linker and Ligand Density on in Vivo siRNA Delivery

**Figure 3:**
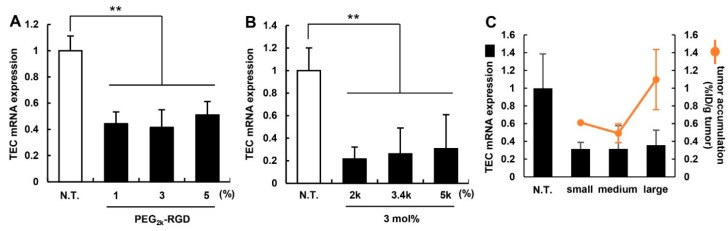
The *in vivo* silencing efficiency of the RGD-modified MEND_vivo_. When the density of the ligand (**A**) and the length of PEG-linker (**B**) were varied, the gene silencing in TECs in murine tumor-bearing mice was measured by qRT-PCR 24 h after the injection of 2.0 mg siRNA/kg body weight of MENDs. Data represent the mean ± S.D. (*n* = 3). (**C**) When the MENDs (small: 98 d.nm, medium: 137 d.nm, large: 282 d.nm in diameter) were systemically administered into mice, the TEC mRNA level and tumor accumulation amount were evaluated by qRT-PCR and fluorescent intensity of the MENDs. The black bar and orange line indicate mRNA expression level and tumor accumulation amount, respectively. **, *p* < 0.01 (one-way ANOVA followed by Bonferroni test).

To evaluate the *in vivo* knockdown ability of the MEND_vivo_ for optimization, murine breast cancer 4T1-bearing mice were used. After the MEND_vivo_ that had been modified with various PEG-lipids was administered to mice via the tail vein, the target mRNA level was measured by qRT-PCR. In this experiment, *Cd31* (also known as *PeCAM*), which is specifically expressed in endothelial cells, was regarded as a target gene. *Cd31* expression in tumor tissue was determined by qRT-PCR at 24 h after mice were treated with RGD-modified MENDs at a dose of 2.0 mg/kg (Figure 3A,B). The tendency for *in vivo* knockdown efficiency was similar to that of *in vitro* knockdown. No drastic change was observed in gene silencing capability. However, 3 mol% and PEG_2k_-RGD appeared to be slightly superior to the others. Therefore, we concluded that the MEND_vivo_ modified with 3 mol% of PEG_2k_-RGD should be used in the following experiments. We then evaluated the effect of the size of the MENDs on *in vivo* gene silencing. The diameter of the MENDs was controlled by changing the amount of PEG-DMG in the preparation. After small (98 nm in diameter; d.nm), medium (137 d.nm), and large (282 d.nm) MENDs were administered to mice, the TEC mRNA expression level and the amount of tumor accumulation for the MENDs were measured. In gene silencing, all of the MENDs were similar, while the amount of tumor accumulation for the large MEND was higher than the others (Figure 3C). However, the difference among the three different lengths of the linkers was not significant. The increased tumor accumulation might be attributed to the increased binding force of cRGD to α_V_β_3_ integrin. We previously revealed that large sized liposomes modified with cRGD showed a higher dissociation constant (K_D_) value [18]. This multivalent interaction succeeded in increasing intratumoral accumulation. In contrast, gene silencing was comparable among them. We assumed that a larger sized nanocarrier was inferior to a smaller sized particle for intracellular trafficking. Further studies will be required to develop a thorough understanding of active targeting delivery.

### 3.4. Evaluation for the Gene Silencing Ability of Optimized MEND_vivo_ in the Human Carcinoma Model

**Figure 4:**
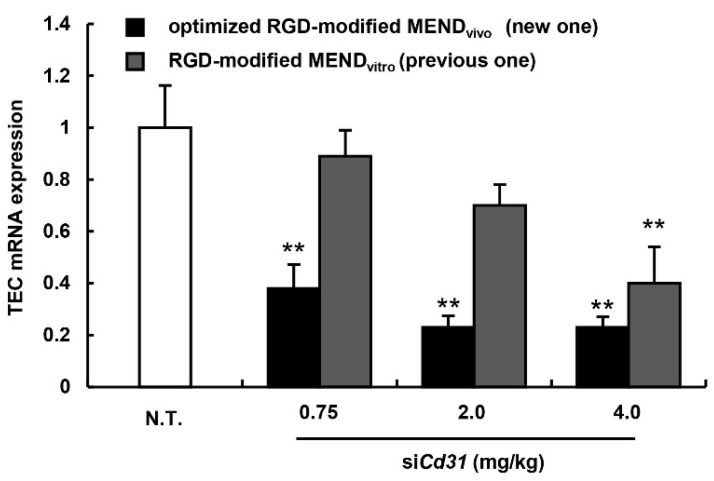
The *in vivo* silencing efficacy of RGD-modified MEND_vivo_. The gene silencing in TECs in murine tumor-bearing mice was measured by qRT-PCR 24 h after the injection of 2.0 mg siRNA/kg body weight of MENDs. Black and gray bars denote the optimized MEND_vivo_ and MEND_vitro_, respectively. Data represent mean ± S.D. (*n* = 3) **, *p* < 0.01 ANOVA followed by SNK test (*vs*. N.T.).

We next compared the optimized RGD-modified MEND_vivo_ (new one) with the RGD-modified MEND_vitro_ (traditional one) in *in vivo* gene silencing ability with human renal cell carcinoma OS-RC-2 cells. As shown in Table 2, the characters of both MENDs are similar. Nude mice were inoculated with OS-RC-2 cells on the right flank, and the mice were then treated with RGD-modified MEND_vivo_ or RGD-modified MEND_vitro_ approximately a week after the inoculation (Figure 4). In the case of the RGD-modified MEND_vitro_, the mRNA level of the TEC-specific gene *Cd31* was decreased only at a dose of 4.0 mg/kg (Figure 4). On the other hand, the RGD-modified MEND_vivo_ exhibited a significant knockdown even at a dose of 0.75 mg/kg. In short, the altered ligand modification method and lipid composition resulted in a five-fold enhancement in the gene silencing ability of RGD-modified MEND. This gene silencing ability would be comparable to the TEC-targeting carrier Atu027, which is now in the clinical stage of testing. Previously, Aleku *et al.* reported that an siRNA lipoplex containing their original cationic lipid exhibited a low ED_50_ (<0.7 mg/kg). Accordingly, our carrier could be equal to the carrier that is now being clinically evaluated for gene silencing efficacy [19].

**Table 2:** Characterization of the MENDs used in the *in vivo* study.

Sample Name	Size (nm)	ZP (mV)	PdI	EE (%)	RR (%)
Optimized RGD-modified MEND_vivo_ (new one)	84 ± 6	−21 ± 14	0.23 ± 0.06	99 ± 2	84 ± 5
RGD-modified MEND_vitro_ (previous one)	92 ± 3	−14 ± 9	0.20 ± 0.03	95 ± 1	88 ± 7

ZP: zeta potential; PdI: polydispersity index; EE: encapsulation efficiency; RR: recovery rate.

**Figure 5:**
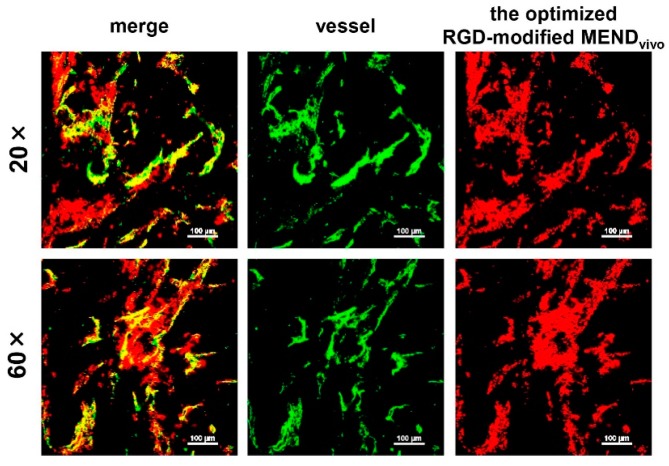
Intratumoral localization of the optimized RGD-modified MEND_vivo_. The MEND was injected into OS-RC-2-bearing mice, and, 24 h after the injection, tumor tissue was excised. The collected tumor tissue was stained with FITC-labeled GSIB4, and observed with CLSM with a 20× objective lens (upper panels) and 60× objective lens (lower panels). Green and red dots represent the vessel and the MEND, respectively.

The intratumoral distribution of the optimized RGD-modified MEND_vivo_ was observed by CLSM. Tumor tissue was observed 24 h after the injection of the fluorescent labeled MEND (Figure 5). The MEND (red) was co-localized in vessels (green), and no diffusion into tumor tissue was detected. This might be because of the strong binding to TECs via cRGD-limited diffusion.

**Figure 6:**
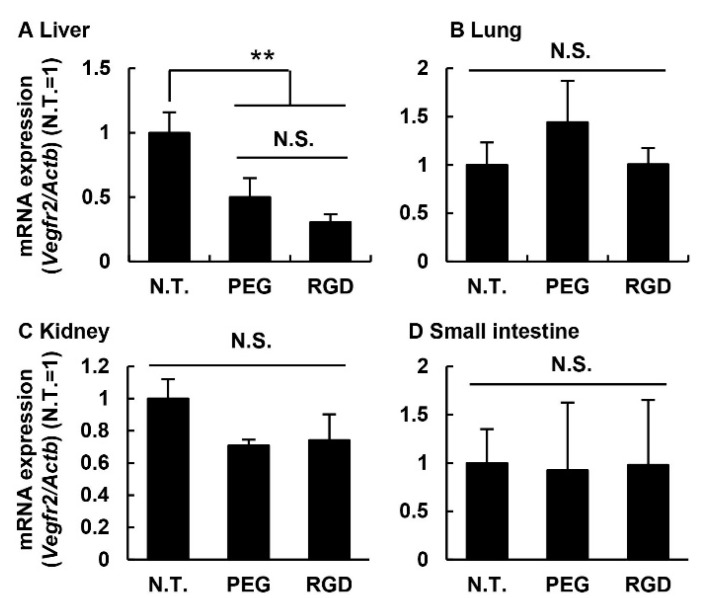
Gene silencing in off-target organs. *Vegfr2* expression levels in the liver, lung, kidney, and small intestine were determined by qRT-PCR 24 h after ICR mice were treated with 3.0 mg/kg of PEG- or RGD-MEND. Data represent mean ± S.D. (*n* = 3). **, *p* < 0.01 (one-way ANOVA followed by Bonferroni test).

### 3.5. Knockdown Efficiency in Off-Target Tissues

To assess the possibility that unpredicted adverse effects could occur in off-target organs, the optimized RGD-modified MEND_vivo_ were systemically administered to ICR mice via the tail vein at a dose of 3.0 mg/kg. One day after the injection, as an endothelial marker, mRNA levels of the VEGF receptor 2 (*Vegfr2*) in the liver, lung, kidney, and small intestine were determined (Figure 6). In the experiment, the absence of the ligand-modified MEND_vivo_ (PEG-MEND) was regarded as a negative control. No gene silencing was observed in the lung, kidney, and small intestine (Figure 6). The lung, kidney, and small intestine are the organs responsible for the adverse effects of anti-angiogenic therapeutics. This result suggests that anti-angiogenic therapy would be a safer system than small molecule inhibitors with respect to organ specificity. In contrast, the level of the endothelial marker was decreased in liver tissue in the case of both the PEG-MEND and the RGD-MEND. In other words, this inhibition did not appear to be mediated by cRGD. Liver endothelial cells are known to develop pinocytosis ability that is involved with the clearance of waste (oxidized low density lipoprotein and oligosaccharide) [20,21]. This property might allow for the non-specific pinocytosis of the two MENDs and, consequently, inhibit the expression of the endothelial marker. However, we injected the optimized RGD-modified MEND_vivo_ three times in one day, one week after the final injection mRNA level of *Vegfr2* was measured (Figure S3). The *Vegfr2* mRNA level of the continuously treated mice was the same as that for the non-treated mice. Accordingly, this inhibition would be transient and, therefore, the inhibition in liver endothelial cells by MENDs might not be a serious issue. Further study will be needed to circumvent this inhibition in off-target organs.

## 4. Conclusions

In this study, we established an efficient methodology for modifying a particle with a PEG-lipid. Based on this methodology, the conditions for PEG-lipid modification, the ligand density, and the length of the ligand were optimized. The optimized RGD-MEND showed an efficiency of 10-fold in gene silencing in tumor endothelial cells, and the ED_50_ was <0.75 mg/kg. From the view point of efficiency, the optimized RGD-MEND represents one of the most efficient siRNA carriers reported to date for targeting tumor endothelial cells.

## Supplementary Files

Supplementary File 1
