# Supplementary material for: Optimization of a siRNA Carrier Modified with a pH-Sensitive Cationic Lipid and a Cyclic RGD Peptide for Efficiently Targeting Tumor Endothelial Cells
Source: Pharmaceutics. 2015 Sep 14;7(3):320–33. doi: 10.3390/pharmaceutics7030320 (PMC4588203; doi:10.3390/pharmaceutics7030320)
Supplement: Supplementary File 1 [file pharmaceutics-07-00320-s001.pdf]

# Supplementary Materials

**Table S1.** The sequences of oligonucleotides used in this study.

| Name | | Sequence (5′ to 3′) |
|---|---|---|
| | | siRNA |
| *PLK1* | sense | aga u*ca ccc u*cc uu*a aau* auu |
| | antisense | uau uua ag*g agg gug au*c uuu |
| *Cd31* | sense | gug cau agu uca agu gac aTT |
| | antisense | ugu cac uug aac uau gca cTT |
| *Vegfr2* | sense | cca gug gac gga uga uca aTT |
| | antisense | uug auc auc cgu cca cug gTT |
| | | primer |
| *PLK1* | forward | CTC CTT GAT GAA GAA GAT CAC C |
| | reverse | GAA GAA GTT GAT CTG CAC GC |
| *GAPDH* | forward | CCT CTG ACT TCA ACA GCG AC |
| | reverse | CGT TGT CAT ACC AGG AAA TGA G |
| *Cd31* | forward | TAC AGT GGA CAC TAC ACC TG |
| | reverse | GAC TGG AGG AGA ACT CTA AC |
| *Vegfr2* | forward | GAT TTC ACC TGG CAC TCT C |
| | reverse | CTT GGT CAC TCT TGG TCA C |
| *Actb* | forward | GAA GGA GAT TAC TGC TCT GG |
| | reverse | ACA CAG AGT ACT TGC GCT CA |

upper case, DNA; lower case, RNA; *, 2′-OMe modification.

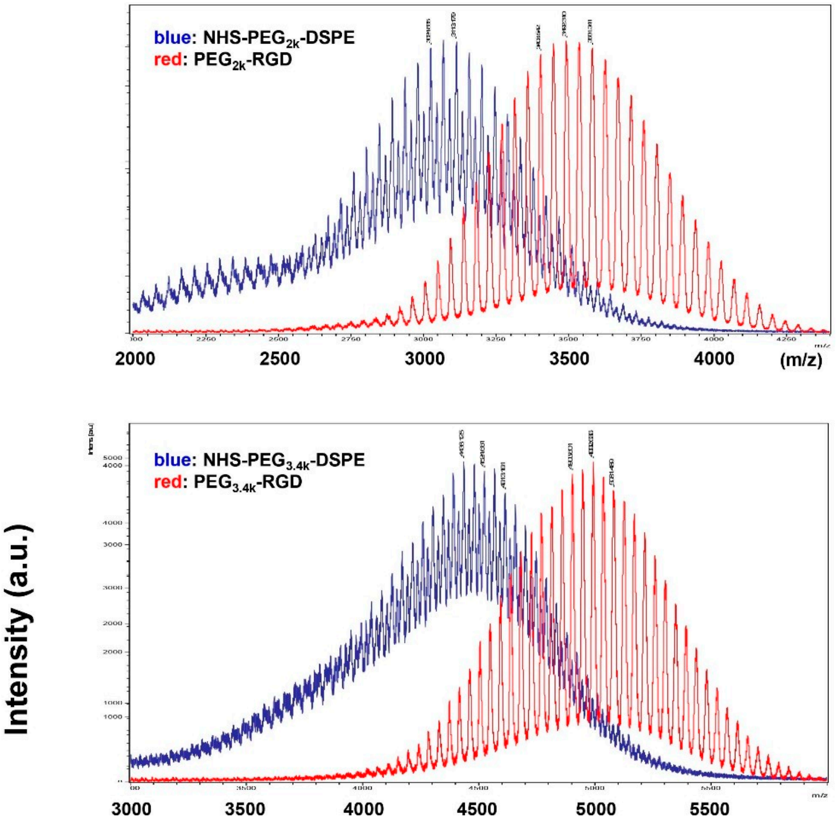


**Figure S1.** *Cont.*

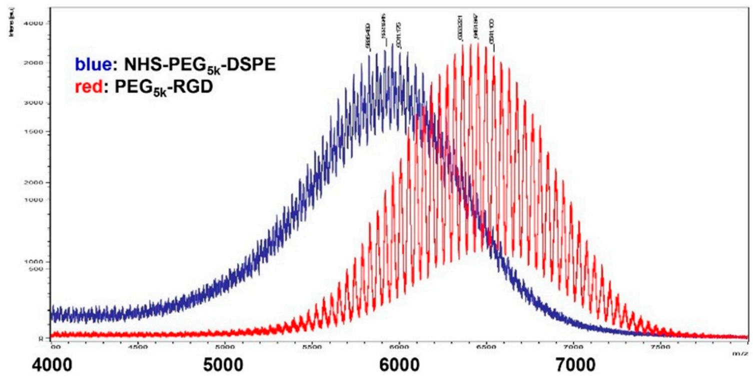


**Figure S1.** The mass spectrum of RGD PEG-lipids. Purified RGD PEG-lipids were subjected to matrix assisted laser desorption ionization time-of-filght mass spectrometry (MALDI TOF-MS).

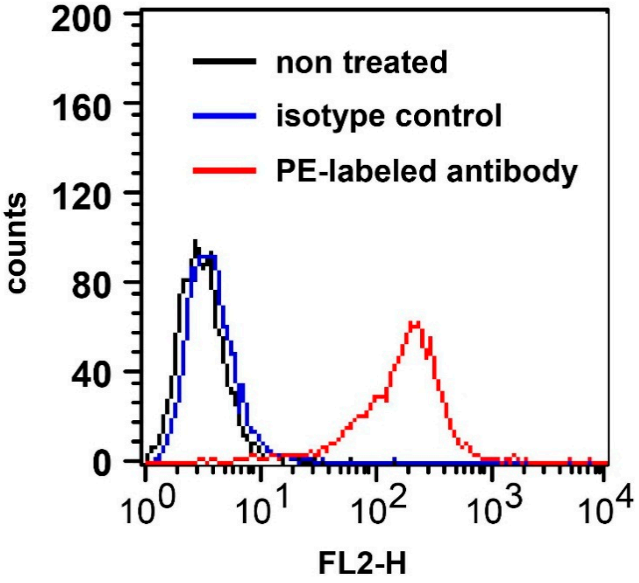


**Figure S2.** The αvβ3 integrin expression of OS-RC-2 cells. To measure protein expression, $1.0 \times 10^5$ trypsinized cells were treated with 1 μg of PE-labled antibody on ice. Then, cells were analyzed by flow cytometry, FACSCalibur (Becton Dickinson). Black, blue and red lines means, non-treated cells, cells treated with isotype control antibody and cells treated with PE-labled antibody against αvβ3 integrin, respectively.

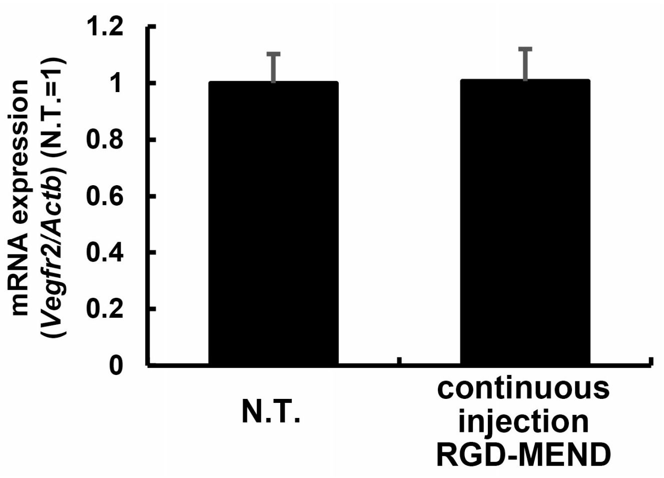


**Figure S3.** The gene silencing 1 week after the injection. The optimized RGD-modified MEND$_{in\ vivo}$ was administered into mice via the tail vein 3 times every a day, and then 1 week after the third administration *Vegfr2* mRNA level was determined by qRT-PCR.

© 2015 by the authors; licensee MDPI, Basel, Switzerland. This article is an open access article distributed under the terms and conditions of the Creative Commons Attribution license (http://creativecommons.org/licenses/by/4.0/).
